# Dietary patterns by cluster analysis in pregnant women: relationship with nutrient intakes and dietary patterns in 7‐year‐old offspring

**DOI:** 10.1111/mcn.12353

**Published:** 2016-10-09

**Authors:** Ana Amélia Freitas‐Vilela, Andrew D. A. C. Smith, Gilberto Kac, Rebecca M. Pearson, Jon Heron, Alan Emond, Joseph R. Hibbeln, Maria Beatriz Trindade Castro, Pauline M. Emmett

**Affiliations:** ^1^ Nutritional Epidemiology Observatory, Department of Social and Applied Nutrition, Institute of Nutrition Josué de Castro Rio de Janeiro Federal University Rio de Janeiro RJ Brazil; ^2^ School of Social and Community Medicine University of Bristol Bristol UK; ^3^ Section of Nutritional Neurosciences, Laboratory of Membrane Biology and Biophysics, National Institute on Alcohol Abuse and Alcoholism National Institutes of Health Bethesda Maryland USA

**Keywords:** dietary patterns, cluster analysis, pregnancy, children, nutritional epidemiology, ALSPAC

## Abstract

Little is known about how dietary patterns of mothers and their children track over time. The objectives of this study are to obtain dietary patterns in pregnancy using cluster analysis, to examine women's mean nutrient intakes in each cluster and to compare the dietary patterns of mothers to those of their children. Pregnant women (*n* = 12 195) from the Avon Longitudinal Study of Parents and Children reported their frequency of consumption of 47 foods and food groups. These data were used to obtain dietary patterns during pregnancy by cluster analysis. The absolute and energy‐adjusted nutrient intakes were compared between clusters. Women's dietary patterns were compared with previously derived clusters of their children at 7 years of age. Multinomial logistic regression was performed to evaluate relationships comparing maternal and offspring clusters. Three maternal clusters were identified: ‘fruit and vegetables’, ‘meat and potatoes’ and ‘white bread and coffee’. After energy adjustment women in the ‘fruit and vegetables’ cluster had the highest mean nutrient intakes. Mothers in the ‘fruit and vegetables’ cluster were more likely than mothers in ‘meat and potatoes’ (adjusted odds ratio [OR]: 2.00; 95% Confidence Interval [CI]: 1.69–2.36) or ‘white bread and coffee’ (OR: 2.18; 95% CI: 1.87–2.53) clusters to have children in a ‘plant‐based’ cluster. However the majority of children were in clusters unrelated to their mother dietary pattern. Three distinct dietary patterns were obtained in pregnancy; the ‘fruit and vegetables’ pattern being the most nutrient dense. Mothers' dietary patterns were associated with but did not dominate offspring dietary patterns.

## Introduction

Energy and nutrient requirements during pregnancy are increased because of maternal metabolic demands to achieve an optimal length of gestation and fetal development (Ramakrishnan *et al*. 2012; Grieger & Clifton 2014). An unhealthy diet and inadequate nutrient intakes during pregnancy may influence weight gain during pregnancy (Maslova *et al*. 2015), hypertensive disorders of pregnancy (Schoenaker *et al*. 2014), maternal (Chatzi *et al*. 2011) and offspring (Jacka *et al*. 2013) mental health, birthweight and fetal growth (Okubo *et al*. 2012; Ramakrishnan *et al*. 2012; Grieger & Clifton 2014).

Adequate nutrient intake during pregnancy is influenced by the consumption of a balanced diet, which needs to be composed of a range of food items providing an adequate supply of macronutrients and bioavailable micronutrients (Black 2001). Dietary patterns have been commonly used in epidemiological studies of diet, as a method of assessing the combination of the usual foods consumed. Dietary patterns describe the habitual diet as an overall dietary exposure, accounting for the fact that people do not eat isolated nutrients or foods (Hu 2002; Newby & Tucker 2004; Wirfält *et al*. 2013). Different approaches have been used to obtain dietary patterns. The principal component analysis (PCA) which is variable‐centred and cluster analysis which is person‐centred are the statistical methods most used. PCA correlates foods or food groups and each individual has a score for all the dietary patterns obtained. Cluster analysis separates individuals into mutually exclusive and non‐overlapping clusters of subjects who consume similar foods (Hu 2002; Newby & Tucker 2004; Bailey *et al*. 2006; Smith *et al*. 2011; Devlin *et al*. 2012; Wirfält *et al*. 2013). This approach allows for a detailed comparison of energy and nutrient intakes between the clusters (Okubo *et al*. 2011).

Dietary patterns may be used to assess the stability of dietary intake or to follow changes in intake over time (Newby & Tucker 2004). The tracking of dietary patterns has been described in the literature, although to our knowledge, never between mothers and their children. Tracking has been observed in different periods of life, during adulthood (Mishra *et al*. 2006), from before to during pregnancy (Crozier *et al*. 2009), from pregnancy to post‐partum (Northstone & Emmett 2008) and from mid‐childhood to early adolescence (Northstone *et al*. 2013). Most of the studies found small changes in dietary patterns over time with the ‘healthy’ diet having greater stability than other types of diet.

There are currently very few studies, in diverse settings, which use cluster analysis to derive dietary patterns during pregnancy (Okubo *et al*. 2011; Okubo *et al*. 2012; Hoffmann *et al*. 2013; McGowan & McAuliffe 2013) and there are no studies that compare dietary patterns between mothers and their children. Cluster analyses previously identified three dietary patterns among children in the ALSPAC cohort (Smith *et al*. 2011). Therefore, the purpose of this study was to obtain dietary patterns in pregnancy using cluster analysis, to examine the mean nutrient intakes of women in each cluster and to compare the dietary patterns of mothers to those of their children.

**Key messages**

- Three dietary patterns during pregnancy were obtained by cluster analysis and labelled as ‘fruit and vegetables’, ‘meat and potatoes’ and ‘white bread and coffee’ clusters.
- Women in the ‘meat and potatoes’ cluster had the highest absolute mean intakes of energy and many nutrients; however, after energy adjustment the highest mean nutrient intakes were observed in women in the ‘fruit and vegetables’ cluster.
- The women in the ‘fruit and vegetables’ cluster during pregnancy were more likely to have children in the ‘plant‐based’ cluster and less likely to have children in the ‘processed’ cluster compared with women in the other two clusters.

## Methods

### Sample

The Avon Longitudinal Study of Parents and Children (ALSPAC) is a longitudinal birth cohort designed to investigate the determinants of health and disease during pregnancy, childhood and beyond (Golding *et al*. 2001; Boyd *et al*. 2013; Fraser *et al*. 2013). Pregnant women residing in the former county of Avon health area in Southwest England and who had an estimated date of delivery between 1 April 1991 and 31 December 1992 were eligible and invited for this study. A total of 14 541 pregnancies were enrolled and 13 988 infants survived to 1 year of age. Ethical approval for the study was granted by ALSPAC Law and Ethics Committee and Local Research Ethics Committees. More details about ALSPAC are available at the website (http://www.bristol.ac.uk/alspac/) which contains details of all the data that are available through a fully searchable data dictionary (http://www.bris.ac.uk/alspac/researchers/data-access/data-dictionary).

### Assessment of dietary intake

The dietary intake was estimated by an unquantified food frequency questionnaire (FFQ) self‐completed at approximately 32 weeks' gestation, covering the main foods consumed in the UK (Rogers & Emmett 1998). The FFQ was composed of 43 food types with five frequency options, which were assigned into weekly frequencies as follows: (1) never or rarely—0 times per week (pw); (2) once in 2 weeks—0.5 times pw; (3) 1–3 times a week—2 times pw; (4) 4–7 times a week—5.5 times pw; and (5) more than once a day—10 times pw. For eight other foods normally consumed daily there were more detailed questions, e.g. number of cups of tea, coffee or milk, number of slices of bread, type of bread (white or other) and milk (full‐fat or other) normally consumed (Rogers & Emmett 1998). Pregnant women with more than 10 missing dietary items were excluded from the analysis; if 10 or less items were missing, it was assumed that the women never consumed that food item (Northstone *et al*. 2008a,2008b). The number of food groups was reduced to 47 by combining some similar groups before the dietary patterns were derived.

### Nutrients

Daily nutrient intakes were estimated from the FFQ using the nutrient content of foods from the fifth edition of McCance and Widdowsons ‘The Composition of Foods’ and supplements (The Royal Society of Chemistry 1988, 1989, 1991a,1991b, 1992a,1992b, 1993) and standard portion sizes (Ministry of Agriculture, Fisheries and Foods 1993). The weekly frequencies were divided by seven to obtain the daily nutrient values (Rogers & Emmett 1998).

### Childhood dietary patterns

From the FFQ administered when the children were aged 7 years cluster analysis had previously been used to derive three patterns describing the child's diet (Smith *et al*. 2011), which were labelled ‘plant‐based’, ‘traditional British’ and ‘processed’. The diets of children in the first cluster were characterized by high intakes of foods such as non‐white bread, fruit, vegetables, meat substitutes, vegetarian foods and fish. In the second cluster children had the highest intakes of full‐fat milk, oat‐based cereals, meat and potatoes. In the last and largest cluster children had the highest intakes of white bread, processed meat, carbonated drinks, squash and snack foods, and the lowest intakes of fruit, vegetables, potatoes, bran and oat‐based cereals.

### Socio‐demographic variables

Socio‐demographic and lifestyle variables were obtained by self‐completed postal questionnaires at 8, 18 and 32 weeks gestation. Maternal education, housing, crowding at home, stressful life events, maternal age, financial difficulty, partner present, maternal smoking in pregnancy, maternal alcohol use in pregnancy, parity, pre‐pregnancy body mass index (BMI) and ethnic origin were the selected variables included in this study. Maternal education was classified as low (no academic examinations or a vocational level training), medium (O level—academic examination usually taken at age 16 years) and high (A level—academic examination usually taken at age 18 years or degree). Financial difficulty was obtained based on a list of five items (food, clothing, heating, rent/mortgage and things for child) with four answers' options of difficulty to afford (very, fairly, some and no). The maximum score that could be obtained was 20, which was categorized as no financial difficulty (score = 0), some (score = 1–5) or many (score = 6 or more). Pre‐pregnancy BMI [weight (kg)/height (m)^2^] was calculated from the self‐reported weight and height at 12 weeks gestation. The cut‐off points proposed by the World Health Organization (WHO 2000) were used to classify the pre‐pregnancy nutritional status: underweight (<18.5 kg/m^2^), normal weight (18.5–24.9 kg/m^2^), overweight (25.0–29.9 kg/m^2^) or obese (≥30.0 kg/m^2^).

### Statistical analysis

Cluster analysis was applied to obtain dietary patterns using 47 standardized food items; 44 of these items were the same as those used in Northstone *et al.* (2008a), and the additional items were full‐fat milk, other milk and alcohol. Some foods, such as tea, coffee, bread and milk were measured on a different scale to the other variables; therefore all the dietary data were standardized by subtracting the mean and dividing by the range for each variable, which is an appropriate method of standardization for cluster analysis (Gnanadesikan *et al*. 1995). Cluster analysis combines individuals into non‐overlapping subgroups (or clusters) in which foods consumed are relatively similar. K‐means clustering is the main cluster analysis method used to obtain dietary patterns (Newby & Tucker 2004). It minimizes the sum of squares of distances between each woman's food intake and the mean of her cluster. The standard k‐means algorithm may not find the correct cluster solution that minimizes this sum of squares the first time that it is run (Everitt *et al*. 2001); therefore, the algorithm was run 100 times, with different starting positions, to find the solution with the smallest sum of squares differences.

The analyses were performed for two to seven clusters, and the best cluster solution was chosen considering the amount of variation explained by the solution, the size and interpretation of each cluster, and the stability of the solution, which was evaluated using linear discriminant analysis.

The socio‐demographic and lifestyle variations between women in each cluster were compared using the chi‐square test. The absolute and energy‐adjusted nutrient intakes were described according to the clusters and ANOVA and the Tukey–Kramer method were used to verify the differences among the nutrients mean. The dietary patterns of the mothers and their children were compared by cross‐tabulating cluster solutions and the proportion of similar clusters between mother and child were calculated. Multinomial logistic regression with odds ratios (OR) was performed to verify the association between maternal and childhood clusters.

All analyses were performed with the use of statistical software package Stata v13.1.

## Results

A total of 12 436 (85.5%) women returned the questionnaire at 32 weeks gestation, and 12 195 (98.1%) of these had complete dietary intake data to obtain the cluster solution.

The three cluster solution was found to give the best fit, explaining 14.9% of the variation in the sample and only a total of 534 women (4.4%) were reclassified to a different cluster when linear discriminant analysis was applied. Fresh fruit, biscuits, chocolate bars, non‐white bread, chocolate and salad were the foods that discriminated most between the clusters.

The first cluster (*n* = 4478) was labelled the ‘fruit and vegetables’ cluster and characterized by women having the highest consumption of non‐white bread, bran‐ and oat‐based breakfast cereals, crispbreads/crackers, poultry, fish, eggs, cheese, meat substitutes, pulses, nuts, potatoes (not fried), pasta, rice, vegetables, fruit, fruit juice, herbal tea, low‐fat milk and alcohol compared with the other clusters (Table 1). The women in this cluster had the lowest intakes of white bread, other breakfast cereal, meat pies, sausages/burgers, fried foods, fried potatoes, roast potatoes, baked beans, cola, tea, coffee, sweets, chocolates, crisps and full‐fat milk.

**Table 1:** Mean (standard deviation) frequency of weekly intake of foods across clusters among 12 195 pregnant women

Cluster	Fruit and vegetables	Meat and potatoes	White bread and coffee	F (*p*‐Value)*
Cluster size	4478	2469	5248	
White bread (slices)	2.8 (5.4) †	**8**.**4** (**8**.**8**)‡	**8**.**6** (**8**.**7**)‡	769 (<0.001)
Non‐white bread (slices)	**13**.**6** (**9**.**6**)†	7.4 (8.6)‡	5.1 (7.0) §	1290 (<0.001)
Bran‐based cereal	**3**.**4** (**2**.**6**)†	2.1 (2.5)‡	1.4 (2.0) §	845 (<0.001)
Oat‐based cereal	**2**.**6** (**2**.**7**)†	1.8 (2.5)‡	1.2 (1.9) §	471 (<0.001)
Other breakfast cereal	1.5 (2.1) †	**3**.**1** (**2**.**7**)‡	2.2 (2.3)§	405 (<0.001)
Biscuits	2.2 (2.0)†	**6**.**4** (**2**.**8**)‡	1.9 (1.6) §	4711 (<0.001)
Crispbreads/crackers	**0**.**7** (**1**.**4**)†	0.5 (1.2)‡	0.3 (0.8) §	138 (<0.001)
Puddings	1.3 (1.5)†	**2**.**0** (**1**.**9**)‡	0.9 (1.1) §	472 (<0.001)
Cakes/buns	1.4 (1.3)†	**2**.**6** (**2**.**1**)‡	1.2 (1.1) §	893 (<0.001)
Poultry	**1**.**8** (**1**.**3**)†	**1**.**9** (**1**.**3**)†	1.5 (1.1) ‡	127 (<0.001)
Red meat	2.3 (1.9)†	**2**.**9** (**2**.**0**)‡	2.1 (1.6) §	184 (<0.001)
Meat pies	0.4 (0.6) †	**0**.**7** (**0**.**9**)‡	0.6 (0.8)§	238 (<0.001)
Offal	0.1 (0.4)†	0.1 (0.5)†	0.1 (0.4)†	0.65 (0.524)
Sausages, burgers	0.4 (0.6) †	**0**.**7** (**0**.**9**)‡	0.7 (0.8)§	221 (<0.001)
Fried foods (e.g. fish, eggs, bacon)	0.4 (0.7) †	**0**.**9** (**1**.**4**)‡	0.7 (1.0)§	285 (<0.001)
Pizza	0.5 (0.7)†	**0**.**6** (**0**.**9**)‡	0.4 (0.7) §	51.0 (<0.001)
Fish	**2**.**5** (**1**.**9**)†	1.9 (1.8)‡	1.5 (1.5) §	364 (<0.001)
Eggs	**1**.**5** (**1**.**3**)†	**1**.**5** (**1**.**4**)†	1.2 (1.2) ‡	90.7 (<0.001)
Cheese	**4**.**0** (**2**.**3**)†	3.6 (2.3)‡	2.3 (1.9) §	742 (<0.001)
Meat substitutes (soya, tofu, etc.)	**0**.**3** (**1**.**0**)†	0.1 (0.9) ‡	0.1 (0.7) ‡	60.7 (<0.001)
Pulses	**0**.**5** (**1**.**0**)†	0.2 (0.7)‡	0.1 (0.5) §	361 (<0.001)
Nuts	**0**.**5** (**1**.**1**)†	0.3 (0.8)‡	0.2 (0.5) §	217 (<0.001)
Fried potatoes (chips)	0.8 (0.9) †	**1**.**7** (**1**.**4**)‡	1.5 (1.2)§	601 (<0.001)
Roast potatoes	0.7 (0.9) †	**1**.**3** (**1**.**1**)‡	1.1 (1.0)§	376 (<0.001)
Potatoes (not fried)	**3**.**2** (**1**.**9**)†	**3**.**3** (**1**.**9**)†	2.3 (1.6) ‡	352 (<0.001)
Pasta	**1**.**5** (**1**.**2**)†	1.1 (1.1)‡	0.9 (1.0) §	446 (<0.001)
Rice	**1**.**5** (**1**.**2**)†	1.1 (1.2)‡	0.9 (1.0) §	377 (<0.001)
Baked beans	1.3 (1.1) †	**1**.**6** (**1**.**3**)‡	1.3 (1.1)§	72.6 (<0.001)
Leafy green vegetables	**2**.**2** (**1**.**7**)†	1.9 (1.5)‡	1.6 (1.2) §	249 (<0.001)
Other green vegetables	**2**.**5** (**1**.**7**)†	2.0 (1.5)‡	1.6 (1.1) §	453 (<0.001)
Carrots	**2**.**6** (**1**.**8**)†	2.2 (1.7)‡	1.6 (1.2) §	487 (<0.001)
Other root vegetables	**1**.**1** (**1**.**3**)†	0.9 (1.2)‡	0.6 (0.9) §	173 (<0.001)
Peas	2.1 (1.5)†	**2**.**2** (**1**.**6**)‡	1.7 (1.2) §	145 (<0.001)
Salad	**3**.**5** (**2**.**4**)†	2.3 (2.0)‡	1.6 (1.4) §	1148 (<0.001)
Fresh fruit	**8**.**2** (**2**.**3**)†	5.8 (3.0)‡	2.9 (2.1) §	5974 (<0.001)
Fruit juice	**5**.**1** (**3**.**9**)†	3.6 (3.6)‡	2.1 (2.6) §	949 (<0.001)
Cola (cups)	0.8 (2.1) †	**1**.**9** (**3**.**4**)‡	**1**.**9** (**3**.**4**)‡	165 (<0.001)
Tea (cups)	19.5 (15.1) †	**22**.**6** (**17**.**7**)‡	21.5 (19.6)§	27.2 (<0.001)
Coffee (cups)	7.2 (10.4) †	9.2 (12.3)‡	**10**.**1** (**13**.**3**)§	69.1 (<0.001)
Herbal tea (cups)	**8**.**9** (**26**.**8**)†	2.9 (15.0) ‡	1.9 (10.8) ‡	175 (<0.001)
Sweets	0.7 (1.3) †	**1**.**9** (**2**.**5**)‡	0.8 (1.4)§	521 (<0.001)
Chocolate	0.9 (1.1) †	**2**.**6** (**2**.**5**)‡	0.9 (1.1) †	1253 (<0.001)
Chocolate bars	1.3 (1.4) †	**4**.**1** (**2**.**7**)‡	1.6 (1.5)§	2154 (<0.001)
Savoury snacks (crisps)	1.4 (1.7) †	**3**.**1** (**2**.**5**)‡	1.9 (2.0)§	606 (<0.001)
Full‐fat milk (l)	0.8 (1.4) †	**1**.**6** (**1**.**8**)‡	**1**.**5** (**1**.**6**)‡	276 (<0.001)
Low‐fat milk (l)	**1**.**8** (**1**.**5**)†	1.4 (1.6)‡	1.0 (1.4) §	307 (<0.001)
Alcohol	**1**.**1** (**2**.**9**)†	0.9 (2.8) † ‡	0.9 (3.0) ‡	4.27 (0.014)

Total variance = 14.9%. The highest and lowest mean frequency in each row are bold and underlined, respectively.

* F values refer to difference between the clusters according to ANOVA F‐test statistic and *p*‐value refers to ANOVA.

† ‡ § Where superscripts differ there is a difference between cluster means *p* < 0.05 (Tukey–Kramer method).

The second cluster with the lowest number of women (*n* = 2469) was labelled the ‘meat and potatoes’ cluster because of the women having the highest consumption of fried potatoes, roast potatoes, potatoes (not fried), poultry, red meat, meat pies and sausages/burgers, in addition to white bread, other breakfast cereal, biscuits, puddings, cakes/buns, fried foods, pizza, eggs, baked beans, peas, cola, tea, sweets, chocolates, savoury snacks and full‐fat milk than the other clusters. This cluster of women had the lowest intake of meat substitutes and herbal tea.

The largest and the last cluster (*n* = 5248) was composed of women with high intakes of white bread, coffee, cola and full‐fat milk, and the lowest intakes of all foods which characterized the ‘fruit and vegetables’ cluster as well as biscuits, puddings, cakes/buns, red meat, pizza, peas and chocolates. It was called the ‘white bread and coffee’ cluster (Table 1).

The main difference in the characteristics of the women with dietary data compared with those without were the percentage with high educational attainment (35.4% vs. 32.3%), with a mortgaged or owned housing (75.7% vs. 56.0%), likely to be in a stable partnership (97.3% vs. 93.5%), age ≥ 30 years (39.3% vs. 25.7%) and with a parity of less than 2 (80.3% vs. 74.1%, respectively) (data not shown). Women in the ‘fruit and vegetables’ cluster were more likely to have high educational attainment, mortgaged or owned housing, a stable partnership, no financial difficulty, be non‐smokers, of older age, of normal weight pre‐pregnancy and nulliparous compared with women in the other clusters. In the ‘meat and potatoes’ cluster women were more likely to be white, to drink alcohol during pregnancy and be underweight pre‐pregnancy. The women who were in the ‘white bread and coffee’ cluster were more likely to have low education, a non‐stable partnership, many financial difficulties, to smoke during pregnancy, be younger, overweight or obese pre‐pregnancy and have had two deliveries or more, when compared with the women who were in the other dietary clusters (Table 2).

**Table 2:** Characteristics of 12 195 pregnant women by cluster

Confounders	Total	Fruit and vegetables	Meat and potatoes	White bread and coffee	*P*‐value*
	*n* (%)	*n* (%)	*n* (%)	*n* (%)	
Maternal education					<0.001
Low	3622 (29.9)	670 (15.0)†	727 (29.6)‡	2225 (42.8)§	
Middle	4210 (34.7)	1319 (29.6)†	972 (39.5)‡	1919 (36.9)§	
High	4292 (35.4)	2474 (55.4)†	761 (30.9)‡	1057 (20.3)§	
Housing					<0.001
Mortgaged/owned	8934 (75.7)	3724 (85.1)†	1875 (78.4)‡	3335 (66.2)§	
Public housing	1508 (12.8)	208 (4.7)†	265 (11.1)‡	1035 (20.6)§	
Other	1360 (11.5)	446 (10.2)†	251 (10.5)‡	663 (13.2)§	
Crowding at home					<0.001
<1 person/room	8725 (75.0)	3630 (83.9)†	1788 (75.6)‡	3307 (66.9)§	
>1 person/room	2905 (25.0)	696 (16.1)†	576 (24.4)‡	1633 (33.1)§	
Financial difficulty					<0.001
None (0)	4356 (35.9)	1992 (44.7)†	848 (34.5)‡	1516 (29.1)§	
Some (1–5)	5346 (44.1)	1843 (41.3)†	1133 (46.2)‡	2370 (45.4)§	
Many (>6)	2429 (20.0)	626 (14.0)†	474 (19.3)‡	1329 (25.5)§	
Life events					0.007
0–7	10 296 (91.8)	3862 (92.8)†	2109 (92.1)† ‡	4325 (90.9)‡	
8–18	915 (8.2)	302 (7.2)†	182 (7.9)† ‡	431 (9.1)‡	
Partner					<0.001
No	326 (2.7)	82 (1.9)†	49 (2.0)†	195 (3.8)‡	
Yes	11 586 (97.3)	4330 (98.1)†	2364 (98.0)†	4892 (96.2)‡	
Maternal age					<0.001
<20 years	464 (3.8)	64 (1.4)†	98 (4.0)‡	302 (5.8)§	
20 – 30 years	6927 (56.9)	2075 (46.4)†	1491 (60.5)‡	3361 (64.1)§	
≥30 years	4787 (39.3)	2333 (52.2)†	876 (35.5)‡	1578 (30.1)§	
Maternal smoking in pregnancy					<0.001
Non‐smoker	5971 (50.4)	2503 (56.9)†	1269 (52.8)‡	2199 (43.5)§	
Stopped	3607 (30.4)	1474 (33.5)†	705 (29.3)‡	1428 (28.3)§	
Still smoking	2275 (19.2)	419 (9.6)†	430 (17.9)‡	1426 (28.2)§	
Maternal alcohol use in pregnancy					<0.001
Non‐drinker	910 (7.7)	246 (5.6)†	177 (7.4)‡	487 (9.7)§	
Stopped	4454 (37.6)	1737 (39. 4)†	856 (35.5)‡	1861 (36.9)§	
Still drinking	6491 (54.7)	2420 (55.0)†	1376 (57.1)‡	2695 (53.4)§	
Parity (number of deliveries)					<0.001
0	5264 (44.8)	2239 (51.3)†	928 (38.9)‡	2097 (42.0)§	
1	4172 (35.5)	1404 (32.2)†	983 (41.3)‡	1785 (35.7)§	
≥2	2307 (19.7)	723 (16. 5)†	472 (19.8)‡	1112 (22.3)§	
Pre‐pregnancy BMI					<0.001
Underweight	520 (4.8)	167 (4.1)†	146 (6.6)‡	207 (4.6)§	
Normal weight	8057 (74.7)	3294 (80.5)†	1667 (75.9)‡	3096 (68.9)§	
Overweight	1624 (15.1)	489 (11.9)†	301 (13.7)‡	834 (18.6)§	
Obese	582 (5.4)	144 (3.52)†	83 (3.8)‡	355 (7.9)§	
Ethnic origin					<0.001
White	11 732 (97.4)	4305 (97.2)†	2405 (98.6)‡	5022 (97.0)†	
Black	127 (1.1)	43 (1.0)†	13 (0.5)‡	71 (1.4)†	
Asian	185 (1.5)	81 (1.8)†	22 (0.9)‡	82 (1.6)†	

* *P*‐values refer to chi‐square test.

† ‡ § Where superscripts differ there is a significant difference among confounder variables according to the clusters.

For absolute mean intakes, women in the ‘white bread and coffee’ cluster had the lowest intake of energy and all nutrients, while those in the ‘meat and potatoes’ cluster had the highest absolute mean intakes of energy and most macronutrients except total n‐3 highly unsaturated fatty acids and fibre and the highest absolute mean intakes of most micronutrients except magnesium, carotene, folate, vitamins C and D (Table 3). Women in the ‘fruit and vegetables’ cluster had the highest absolute mean intakes of docosahexaenoic acid (DHA), eicosapentaenoic acid (EPA) and total n‐3 highly unsaturated fatty acids, fibre, magnesium, carotene, folate, vitamin C and D (Table 3). After energy adjustment, the mean intakes of almost all of the nutrients were higher in the ‘fruit and vegetables’ cluster showing that this was the most nutrient dense dietary pattern. Conversely the ‘white bread and coffee’ pattern was the least nutrient dense (Table 4).

**Table 3:** Mean (standard deviation) of daily absolute nutrient intakes according to clusters of dietary patterns among 12 195 pregnant women

Nutrients	Total	Fruit and vegetables	Meat and potatoes	White bread and coffee	*P*‐value*
	Mean (SD)	Mean (SD)	Mean (SD)	Mean (SD)	
Energy (MJ)	7.24 (2.01)	7.27 (1.69)†	9.02 (2.02)‡	6.39 (1.69)§	<0.001
Carbohydrate (g)	212 (62.8)	214 (50.7)†	268 (63.3)‡	186 (54.2)§	<0.001
Protein (g)	69.4 (19.7)	75.0 (18.2)†	79.6 (19.3)‡	59.9 (16.7)§	<0.001
Fat (g)	71.8 (23.4)	69.3 (20.5)†	91.4 (24.2)‡	64.7 (20.0)§	<0.001
Monounsaturated fat (g)	24.2 (7.99)	23.3 (7.00)†	31.0 (8.30)‡	21.7 (6.80)§	<0.001
Polyunsaturated fat (g)	12.3 (4.58)	13.0 (4.49)†	14.4 (4.64)‡	10.7 (4.06)§	<0.001
Saturated fat (g)	30.1 (11.5)	27.9 (9.96)†	39.7 (12.1)‡	27.5 (10.1)†	<0.001
n‐3 fatty acid (g)	0.15 (0.15)	0.19 (0.16)†	0.14 (0.15)‡	0.11 (0.13)§	<0.001
DHA (g)	0.07 (0.07)	0.09 (0.08)†	0.06 (0.07)‡	0.05 (0.06)§	<0.001
EPA (g)	0.05 (0.05)	0.07 (0.05)†	0.05 (0.05)‡	0.04 (0.04)§	<0.001
Sugar (g)	96.0 (39.0)	92.8 (27.5)†	130.7 (41.4)‡	82.5 (36.4)§	<0.001
Free sugar (g)	60.6 (34.6)	51.0 (23.0)†	91.9 (38.1)‡	54.1 (32.7)§	<0.001
Fibre (g)	14.9 (5.13)	18.0 (4.70)†	16.4 (4.44)‡	11.5 (3.51)§	<0.001
Calcium (mg)	939 (287)	991 (263)†	1093 (289)‡	823 (258)§	<0.001
Iron (mg)	10.2 (3.32)	11.8 (3.02)†	11.7 (3.09)†	8.2 (2.48)‡	<0.001
Zinc (mg)	8.17 (2.38)	9.01 (2.18)†	9.35 (2.30)‡	6.91 (1.94)§	<0.001
Sodium (mg)	2195 (647)	2322 (605)†	2541 (645)‡	1925 (569)§	<0.001
Magnesium (mg)	247 (74.6)	285 (67.9)†	280 (67.5)‡	199 (53.4)§	<0.001
Potassium (mg)	2882 (738)	3061 (652)†	3315 (725)‡	2528 (644)§	<0.001
Retinol (µg)	367 (362)	353 (319)†	432 (380)‡	349 (384)†	<0.001
Carotene (µg)	2133 (1177)	2578 (1292)†	2279 (1173)‡	1686 (876)§	<0.001
Riboflavin (mg)	1.70 (0.56)	1.80 (0.51)†	1.99 (0.58)‡	1.48 (0.50)§	<0.001
Folate (µg)	243 (73.4)	279 (66.2)†	273 (68.3)‡	199 (55.7)§	<0.001
Iodine (µg)	148 (48.5)	157 (46.3)†	172 (48.4)‡	129 (42.9)§	<0.001
Niacin (mg)	16.0 (5.19)	17.9 (4.87)†	18.1 (5.05)†	13.3 (4.25)‡	<0.001
Thiamine (mg)	1.43 (0.42)	1.60 (0.39)†	1.63 (0.40)‡	1.19 (0.33)§	<0.001
Vitamin B6 (mg)	1.89 (0.54)	2.07 (0.49)†	2.17 (0.53)‡	1.60 (0.44)§	<0.001
Vitamin B12 (µg)	4.88 (2.70)	5.38 (2.66)†	5.33 (2.76)†	4.24 (2.57)‡	<0.001
Vitamin C (mg)	79.7 (35.3)	103 (32.0)†	84.6 (31.4)‡	57.1 (23.2)§	<0.001
Vitamin D (µg)	3.83 (2.11)	4.46 (2.23)†	4.08 (2.08)‡	3.17 (1.81)§	<0.001
Vitamin E (mg)	8.54 (4.12)	9.99 (4.16)†	9.84 (4.02)†	6.69 (3.33)‡	<0.001

* *P* value refers to ANOVA test.

† ‡ § Where superscripts differ there is a difference between cluster means *p* < 0.05 (Tukey–Kramer method and Kruskal–Wallis tests).

**Table 4:** Mean (standard deviation) of daily nutrient intakes adjusting for energy intakes according to clusters dietary patterns among 12 195 pregnant women

Nutrients	Fruit and vegetables	Meat and potatoes	White bread and coffee	*P*‐value*
	Mean (SD)	Mean (SD)	Mean (SD)	
Carbohydrate (g)	213 (21.3)†	216 (24.4)‡	211 (22.3)§	<0.001
Protein (g)	74.8 (10.3)†	65.4 (12.21)‡	66.8 (10.1)§	<0.001
Fat (g)	69.1 (8.34)†	72.2 (9.19)‡	73.9 (8.32)§	<0.001
Monounsaturated fat (g)	23.2 (3.01)†	24.5 (3.35)‡	24.9 (3.03)§	<0.001
Polyunsaturated fat (g)	12.9 (3.25)†	11.6 (3.48)‡	12.1 (3.15)§	<0.001
Saturated fat (g)	27.8 (5.94)†	31.1 (6.48)‡	31.6 (5.82)§	<0.001
n‐3 fatty acid (g)	0.19 (0.16)†	0.11 (0.14)‡	0.12 (0.12)§	<0.001
DHA (g)	0.09 (0.08)†	0.05 (0.07)‡	0.06 (0.06)§	<0.001
EPA (g)	0.07 (0.05)†	0.04 (0.05)‡	0.04 (0.04)§	<0.001
Sugar (g)	92.4 (20.9)†	104 (28.6)‡	94.9 (27.2)§	<0.001
Free sugar (g)	50.7 (19.1)†	71.5 (28.8)‡	63.9 (26.3)§	<0.001
Fibre (g)	18.0 (3.57)†	13.6 (3.48)‡	12.9 (2.87)§	<0.001
Calcium (mg)	988 (167)†	891 (182)‡	920 (166)§	<0.001
Iron (mg)	11.7 (2.00)†	9.54 (2.10)‡	9.22 (1.83)§	<0.001
Zinc (mg)	8.98 (1.20)†	7.65 (1.45)‡	7.72 (1.21)§	<0.001
Sodium (mg)	2315 (305)†	2049 (358)‡	2161 (307)§	<0.001
Magnesium (mg)	284 (40.5)†	229 (41.7)‡	223 (34.3)§	<0.001
Potassium (mg)	3053 (390)†	2780 (444)‡	2786 (390)‡	<0.001
Retinol (µg)	351 (302)†	330 (352)‡	398 (369)§	<0.001
Carotene (µg)	2574 (1258)†	1993 (1146)‡	1824 (859)§	<0.001
Riboflavin (mg)	1.80 (0.37)†	1.64 (0.42)‡	1.65 (0.38)‡	<0.001
Folate (µg)	278 (45.8)†	227 (49.7)‡	221 (41.2)§	<0.001
Iodine (µg)	156 (33.0)†	140 (32.7)‡	144 (30.1)§	<0.001
Niacin (mg)	17.8 (3.54)†	15.0 (4.03)‡	14.8 (3.35)§	<0.001
Thiamine (mg)	1.60 (0.24)†	1.34 (0.28)‡	1.33 (0.22)§	<0.001
Vitamin B6 (mg)	2.06 (0.32)†	1.81 (0.38)‡	1.78 (0.31)§	<0.001
Vitamin B12 (µg)	5.36 (2.42)†	4.27 (2.44)‡	4.75 (2.34)§	<0.001
Vitamin C (mg)	103 (30.4)†	73.5 (30.2)‡	62.4 (22.7)§	<0.001
Vitamin D (µg)	4.45 (2.03)†	3.30 (1.89)‡	3.55 (1.67)§	<0.001
Vitamin E (mg)	9.96 (3.54)†	7.93 (3.50)‡	7.61 (3.07)§	<0.001

* *P* value refers to ANOVA test.

† ‡ § Where superscripts differ there is a difference between cluster means *p* < 0.05 (Tukey–Kramer method and Kruskal–Wallis tests).

There were 7874 children with dietary pattern data available to compare to their mothers, representing 95.2% (7874/8274) of dietary data of children at 7 years of age and 64.6% (7874/12 195) of eligible pregnancy data. Foods associated with the maternal ‘fruit and vegetables’ cluster were very similar to those in the childhood ‘plant‐based’ cluster. Some foods that characterized the ‘meat and potatoes’ cluster during pregnancy characterized the ‘traditional British’ cluster in childhood. Foods characterizing the ‘processed’ cluster in childhood were a mixture of foods from both the ‘meat and potatoes’ and ‘white bread and coffee’ clusters during pregnancy. The ‘processed’ cluster dominated the diets of the children, whichever cluster the mothers were in during pregnancy. Overall more than half the children were in the ‘processed’ cluster; nevertheless, mother's dietary clusters were associated with some variations in children's dietary clusters (Table 5). Among women who were in the ‘fruit and vegetables’ cluster during pregnancy a third of their children were in the ‘plant‐based’ cluster; in contrast, for mothers in the other two clusters less than a fifth of their children were in the ‘plant‐based’ cluster. Among women in the ‘meat and potatoes’ and ‘white bread and coffee’ clusters, more than half of their children were in the ‘processed’ cluster; in contrast, for mothers in the 'fruit and vegetables' cluster only two fifths of their children were in the ‘processed’ cluster. For all three maternal diet clusters approximately one quarter of the children were in the ‘traditional British’ cluster (Table 5).

**Table 5:** Cross‐tabulation comparing cluster membership of mothers during pregnancy to cluster membership of their children at 7 years of age

	Maternal dietary pattern*							
	Fruit and vegetables*		Meat and potatoes*		White bread and coffee*		Total	
Child's dietary pattern								
	*n*	%	*N*	%	*N*	%	*n*	%
Plant‐based*	1134	35.3	322	19.4	517	17.3	1973	25.1
Traditional British*	743	23.1	445	26.7	738	24.6	1926	24.4
Processed*	1338	41.6	896	53.9	1741	58.1	3975	50.5
Total	3215	100.0	1663	100.0	2996	100.0	7874	100.0

* Chi‐square test <0.001 between maternal and children clusters dietary patterns.

The multinomial logistic regression found associations between maternal and childhood dietary patterns with similar foods (Table 6). Children of women in the ‘fruit and vegetables’ cluster were more than twice, and almost three times as likely to be in the ‘plant‐based’ cluster, than the ‘traditional British’ and ‘processed’ clusters, as compared with children of mothers in the ‘meat and potatoes’ or ‘white bread and coffee’ clusters, respectively. Children of women in the ‘meat and potatoes’ cluster were twice as likely, and only slightly more likely, to be in the ‘traditional British’ cluster, than the ‘fruit and vegetables’ and ‘processed’ clusters, as compared with children of mothers in the ‘fruit and vegetable’ and ‘white bread and coffee’ clusters, respectively. Children of women in the ‘white bread and coffee’ cluster were almost three times as likely to be in the ‘processed’ cluster, than the ‘fruit and vegetables’ and ‘traditional British’ clusters, as compared with children of mothers in the ‘fruit and vegetables’ cluster. These associations were slightly attenuated after adjustment for confounding variables (Table 6).

**Table 6:** Association between child and maternal dietary patterns estimated by unadjusted and adjusted multinomial logistic regression

	Maternal dietary patterns					
	Estimates using fruit and vegetable outcome category as reference		Estimates using meat and potatoes outcome category as reference		Estimates using white bread and coffee outcome category as reference	
Child's dietary patterns	Meat and potatoes	White bread and coffee	Fruit and vegetables	White bread and coffee	Fruit and vegetables	Meat and potatoes
Unadjusted	OR (95% CI)†	OR (95% CI)†	OR (95% CI)†	OR (95% CI)†	OR (95% CI)†	OR (95% CI)†
Plant‐based	1.00 (reference)	1.00 (reference)	1.00 (reference)	1.00 (reference)	1.00 (reference)	1.00 (reference)
Traditional British	2.11 (1.78, 2.50)	2.18 (1.88, 2.52)	0.47 (0.40, 0.56)	1.03 (0.86, 1.24)	0.46 (0.40, 0.53)	0.97 (0.81, 1.16)
Processed	2.36 (2.03, 2.74)	2.85 (2.52, 3.24)	0.42 (0.36, 0.49)	1.21 (1.03, 1.42)	0.35 (0.31, 0.40)	0.83 (0.70, 0.97)
Traditional British	1.00 (reference)	1.00 (reference)	1.00 (reference)	1.00 (reference)	1.00 (reference)	1.00 (reference)
Processed	1.12 (0.97, 1.29)	1.31 (1.16, 1.48)	0.89 (0.77, 1.03)	1.17 (1.02, 1.35)	0.76 (0.67, 0.86)	0.85 (0.74, 0.98)
Plant‐based	0.47 (0.40, 0.56)	0.46 (0.40, 0.53)	2.11 (1.78, 2.50)	0.97 (0.81, 1.16)	2.18 (1.88, 2.52)	1.03 (0.86, 1.24)
Processed	1.00 (reference)	1.00 (reference)	1.00 (reference)	1.00 (reference)	1.00 (reference)	1.00 (reference)
Plant‐based	0.42 (0.36, 0.49)	0.35 (0.31, 0.40)	2.36 (2.03, 2.74)	0.83 (0.70, 0.97)	2.85 (2.52, 3.24)	1.21 (1.03, 1.42)
Traditional British	0.89 (0.77, 0.49)	0.76 (0.67, 0.86)	1.12 (0.97, 1.29)	0.85 (0.74, 0.98)	1.31 (1.16, 1.48)	1.17 (1.02, 1.35)
Adjusted‡	OR (95% CI)†	OR (95% CI)†	OR (95% CI)†	OR (95% CI)†	OR (95% CI)†	OR (95% CI)†
Plant‐based	1.00 (reference)	1.00 (reference)	1.78 (1.47, 2.15)	1.06 (0.88, 1.30)	2.18 (1.87, 2.53)	1.09 (0.91, 1.30)
Traditional British	1.78 (1.47, 2.15)	1.67 (1.41, 1.99)	1.00 (reference)	1.00 (reference)	1.30 (1.12, 1.51)	1.16 (0.99, 1.36)
Processed	2.00 (1.69, 2.36)	2.18 (1.87, 2.53)	0.89 (0.76, 1.04)	1.16 (0.99, 1.36)	1.00 (reference)	1.00 (reference)

† Odds ratios and 95 % confidence intervals.

‡ The analysis was adjusted by maternal education, housing, crowding at home, stressful life events, maternal age, financial difficulty, partner, maternal smoking and alcohol use in pregnancy, parity, pre‐pregnancy BMI, ethnic origin and gender. The reference category of models presented are those with similarity between maternal and child clusters.

## Discussion

Three dietary patterns during pregnancy were obtained by cluster analysis in this cohort of women and labelled as ‘fruit and vegetables’, ‘meat and potatoes’ and ‘white bread and coffee’ clusters. Women in the ‘meat and potatoes’ cluster had the highest absolute mean intakes of energy and many nutrients; however, after energy adjustment the highest mean nutrient intakes were observed in women in the ‘fruit and vegetables’ cluster. The women who were in the ‘fruit and vegetables’ cluster during pregnancy were more likely to have children in the ‘plant‐based’ cluster and less likely to have children in the ‘processed’ cluster compared with women who were in the other two clusters during pregnancy. Even so the largest proportion of children were in the ‘processed’ cluster whichever cluster their mothers had been in during pregnancy.

The most important limitation of this study was the loss of subjects during the follow‐up which is a common problem in cohort studies. We observed only a small loss of data during the pregnancy [16.1% (12 195/14 541) and of these 526 were miscarriages prior to completion of the FFQ]; however, the loss to follow‐up was lower than other cohort studies (Okubo *et al*. 2012; McGowan & McAuliffe 2013; Vilela *et al*. 2014). The FFQ is the instrument most used in epidemiological studies of diet, but memory bias, social desirability of consuming certain foods and under‐ or over‐estimation of consumption frequency by untrained individuals are common in this instrument. Women were asked to report their current food intake to minimize recall bias. There was no portion size information collected in the FFQ; therefore, standard portion sizes were used to estimate nutrient intakes. Thus nutrient estimates were totally reliant on the accuracy of the frequency data. Moreover, considering that the child's FFQ was, for the most part, completed by the mother herself, this may have biassed her reporting.

The present study had important strengths such as large sample size, the use of cluster analysis to obtain dietary patterns which allowed each individual to be categorized exclusively into one dietary pattern, the availability of estimates of energy and nutrient intakes and the cohort design which made it possible to compare the dietary intakes of mother and child pairs over a long period of time.

Northstone *et al*. (2008a) obtained dietary patterns using PCA in this sample of pregnant women. They identified five dietary patterns named; ‘health conscious’, ‘traditional’, ‘processed’, ‘confectionery’ and ‘vegetarian’. The foods characterizing the ‘health conscious’ and ‘vegetarian’ components were consumed frequently in the ‘fruit and vegetables’ cluster of the present study, while foods characterizing the other three components were consumed frequently in the ‘meat and potatoes’ cluster. The foods which composed the ‘white bread and coffee’ cluster (except white bread) had low factor loading in the PCA; thus, they did not contribute to the components in the Northstone *et al*. (2008a) study.

Of the few studies which derived dietary patterns using cluster analysis during pregnancy, only Hoffmann *et al*. (2013) in Brazil used an FFQ to measure the dietary intake. Other studies used a diet history questionnaire in Japan (Okubo *et al*. 2011; Okubo *et al*. 2012) or a 3‐day food diary in Ireland (McGowan & McAuliffe 2013). The three clusters identified by Hoffmann *et al*. (2013) were related to Brazilian dietary habits therefore were substantially different from those among British women. However, in one cluster there were high intakes of fish, fruits and vegetables similar to our ‘fruit and vegetables’ cluster. Okubo *et al*. (2011) obtained three clusters among pregnant Japanese women; again in one cluster there were high intakes of fruits, vegetables and fish. This was the only cluster characterized by foods similar to the current study. In contrast, McGowan & McAuliffe (2013) identified two clusters in the diets among Irish women; one cluster with similarities to our ‘meat and potatoes’ cluster; the second consistent with the ‘fruit and vegetables’ cluster of our study. Furthermore, Crozier *et al*. (2006) obtained two dietary patterns by cluster analysis among young non‐pregnant women from Southampton, UK. One was based on consumption of foods similar to a combination of our ‘meat and potatoes’ and ‘white bread and coffee’ clusters; the second was similar to the ‘fruit and vegetables’ cluster of our study. This research group also investigated changes in diet from before to during pregnancy using PCA derived dietary patterns; they found that the pattern scores changed very little between before and either early or late pregnancy (Crozier *et al*. 2009). Furthermore the group explored whether similar dietary patterns were obtained using diet records rather than an FFQ to assess the diets of women; again using PCA, they found that the two methods defined very similar dietary patterns (Crozier *et al*. 2008). These results suggest that diets among young British and Irish women have similarities, and are not greatly affected by pregnancy.

The nutritional profile of the diet of the current cohort of pregnant women was previously reported (Rogers & Emmett 1998), as well as the correlation between nutrient intakes and dietary patterns obtained by PCA (Northstone *et al*. 2008b). The ‘meat and potatoes’ cluster was characterized by food groups with higher energy‐densities, when compared with the ‘fruit and vegetables’ and ‘white bread and coffee’ clusters, so this cluster had the highest mean energy intake and absolute intake of many nutrients. A similar dietary pattern to our ‘meat and potatoes’ pattern was derived by PCA in pregnant women of Pregnancy, Infection and Nutrition (PIN) Study in North Carolina; the highest quartile of this pattern was associated with a higher energy intake than the highest quartile of a pattern characterized by foods similar to our ‘fruit and vegetables’ cluster (Martin *et al*. 2015).

Nutrient intakes adjusted for energy intake in each cluster showed different results when compared with absolute nutrient intakes. The highest mean intakes of highly unsaturated omega‐3 fatty acids, fibre and most micronutrients were observed in the ‘fruit and vegetables’ cluster; however, mean intakes of some less desirable macronutrients were lowest in this cluster (total fat, monounsaturated and saturated fat, carbohydrate and free sugars). Similarly Northstone *et al*. (2008b) reported that high scores on the PCA ‘processed’ and ‘confectionery’ patterns were associated with higher intakes of sugars and fats after energy adjustment. In Okubo *et al*. (2011) the observed median of daily energy‐adjusted nutrient intakes in their ‘fruits, vegetables and fish’ cluster was similar to that in the ‘fruit and vegetables’ cluster in our study.

To the best of our knowledge, this is the first study of dietary patterns, derived by cluster analysis, comparing women during pregnancy to their offspring in childhood. We observed that although a child's dietary cluster was associated with the mother's dietary cluster during pregnancy, the ‘processed’ dietary cluster dominated the children's diets whatever type of diet the mother had eaten in pregnancy. This suggests that some mothers who eat ‘healthy’ foods themselves none the less feed their children less healthy foods or perhaps that some mothers change their own diets towards more ‘processed’ foods once they have children in the family. ALSPAC has investigated the change in maternal dietary pattern, by PCA, comparing pregnancy to 4 years after the birth of the study child (Northstone & Emmett 2008). There was moderate agreement between the patterns derived at the two time points and the mean score on the ‘healthy’ pattern decreased while that on the ‘processed’ pattern increased confirming a drift towards a more processed dietary pattern. Furthermore, an investigation of preschool diet in ALSPAC children found that intakes of free sugars rose substantially comparing 1.5 and 3.5 years (Emmett *et al*. 2002) suggesting that the profile of consumed foods changes in early childhood. A later study in ALSPAC children, using cluster analysis with diet diaries at 7, 10 and 13 years of age, placed children into four dietary clusters at each age; again the ‘processed’ cluster contained the largest number of children. The ‘healthy’ cluster and the ‘processed’ cluster were the most stable over time suggesting that these types of diet consumed by children at younger ages are likely to track at least over childhood (Northstone *et al*. 2013).

In Southampton Women's Survey diet was obtained when the women were not pregnant (Crozier *et al*. 2006) and a prudent dietary pattern was identified by PCA. Among the offspring, associations were explored comparing maternal and family factors and quality of pre‐school children's diet and the quality of the mother's diet. Higher scores on a maternal prudent diet were the most important determinant of the children's prudent diet scores (Fisk *et al*. 2011). Similarities in cluster dietary patterns over time were observed among adult subjects from the Netherlands who were followed for 6 months: 66.2% of them remained in the same cluster, 11.9% changed to an unhealthier cluster, while 21.9% moved to a healthier cluster (Walthouwer *et al*. 2014).

In conclusion, we obtained three dietary patterns during pregnancy by cluster analysis. Diets of women in the ‘fruit and vegetables’ cluster showed a better nutrient profile after energy adjustment compared with those of women in the ‘meat and potatoes’ and ‘white bread and coffee’ clusters. The type of diet consumed by the mother during pregnancy was associated with, but not an absolute determinant of the type of diet consumed by her offspring in childhood. This study adds to the evidence that improving the diets, or diet education, of pregnant women could potentially help improve the dietary habits of their children. However, it is likely that support will be needed to maintain any improvement in maternal diet and to enhance any beneficial influence on offspring diet.

## Source of funding

The UK Medical Research Council and the Wellcome Trust (Grant ref: 102215/2/13/2) and the University of Bristol provide core support for ALSPAC. PME has received research funding from Wyeth Nutrition and currently receives research funding from Nestle' Nutrition. The study was supported by the Carlos Chagas Filho Foundation for Research Support of Rio de Janeiro State (FAPERJ in the Portuguese cronym). AAFV received a scholarship from the Brazilian Coordination Body for the Training of University Level Personnel (CAPES) (Grant ref: 99999.010031/2014‐06). GK received a scholarship from CNPq (Grant ref: 304182/2013‐3). The intramural program of the National Institute on Alcohol Abuse and Alcoholism provided support for this project. This publication is the work of the authors, and PME will serve as guarantors for the content of this paper.

## Conflicts of interest

The authors declare that they have no conflicts of interest.

## Contributions

Substantial contributions to conception and design of, or acquisition of data or analysis and interpretation of data: AAFV; ADACS; GK; JH; PME. Drafting the article or revising it critically for important intellectual content: AAFV; ADACS; GK; RMP; JH; AE; JRH; MBTC; PME. Final approval of the version to be published: AAFV; ADACS; GK; RMP; JH; AE; JRH; MBTC; PME.
